# Melatonin’s Role in Hair Follicle Growth and Development: A Cashmere Goat Perspective

**DOI:** 10.3390/ijms26072844

**Published:** 2025-03-21

**Authors:** Zibin Zheng, Zhenyu Su, Wei Zhang

**Affiliations:** State Key Laboratory of Animal Nutrition and Feeding, College of Animal Science and Technology, China Agricultural University, Beijing 100193, China; zhengzibin@cau.edu.cn (Z.Z.); suzhenyu@cau.edu.cn (Z.S.)

**Keywords:** melatonin, cashmere growth, hair follicle growth and development, cashmere goat

## Abstract

Hair follicles, unique skin appendages, undergo cyclic phases (anagen, catagen, telogen) governed by melatonin and associated molecular pathways. Melatonin, synthesized in the pineal gland, skin, and gut, orchestrates these cycles through antioxidant activity and signaling cascades (e.g., Wnt, BMP). This review examines melatonin’s biosynthesis across tissues, its regulation of cashmere growth patterns, and its interplay with non-coding RNAs and the gut–skin axis. Recent advances highlight melatonin’s dual role in enhancing antioxidant capacity (via Keap1-Nrf2) and modulating gene expression (e.g., *Wnt10b*, *CTNNB1*) to promote hair follicle proliferation. By integrating multi-omics insights, we construct a molecular network of melatonin’s regulatory mechanisms, offering strategies to improve cashmere yield and quality while advancing therapies for human alopecia.

## 1. Introduction

Hair, a derivative of mammalian skin, originates from hair follicles in the dermis and serves functions such as insulation, protection, communication, and sensory perception. For instance, cashmere fibers in goats provide superior insulation due to their fine diameter (12–19 µm), whereas guard hairs in other mammals (e.g., sheep) are coarser and serve protective roles [1,2]. The hair follicle, the structural unit for hair production, governs hair initiation, growth, shedding, and regeneration via its cyclic activity [3]. Cashmere, one of the earliest natural fibers utilized by humans, is widely valued in the wool spinning industry for its dyeability, fine texture, lightweight properties, and elasticity. It also represents a key economic trait in cashmere goats, with its yield and quality directly influencing the economic outcomes for farmers [4]. The structural complexity of hair follicles directly influences fiber traits; in cashmere goats, secondary hair follicles (SHFs) produce fine undercoat fibers, while primary follicles (PHFs) generate coarse guard hairs. SHFs responsible for producing cashmere undergo distinct phases of anagen, catagen, and telogen, with the development and apoptosis of SHF at each stage regulated by numerous genes [5]. The periodic growth and development of skin hair follicles constitute a complex process involving various cell types and signaling pathways, which is orchestrated by the seasonal secretion of melatonin [6,7]. This cyclical process is tightly regulated by melatonin, a hormone historically recognized for circadian rhythm regulation but now understood to exert multifaceted roles in peripheral tissues. Notably, melatonin directly modulates hair follicle cycling by synchronizing the anagen, catagen, and telogen phases with seasonal photoperiod changes, as observed in cashmere goats [7,8].

Melatonin (*N*-acetyl-5-methoxytryptamine), initially extracted and identified from the bovine pineal gland in 1959 [9], was long considered solely a neurohormone localized within the mammalian central nervous system, primarily involved in regulating circadian rhythms [10,11]. Recent studies highlight its roles in synchronizing peripheral tissue rhythms [12,13], regulating the seasonal biological activities of mammals [14], anti-oxidative stress and anti-aging [15,16], cell proliferation and differentiation [17], and balancing glucose, protein, and lipid metabolism [18,19]. In mammals, melatonin is synthesized via two distinct pathways: (1) the pineal gland (Source 1; less than 5% of melatonin is synthesized by the pineal gland), which rhythmically releases melatonin into the bloodstream and cerebrospinal fluid to regulate circadian and circannual rhythms, and (2) extrapineal tissues (Source 2), where melatonin is constitutively produced in mitochondria to locally modulate redox homeostasis and metabolic processes [20,21,22]. Over the past 40 years, numerous studies have demonstrated that both light exposure and exogenously administered melatonin enhance cashmere production in cashmere goats by modulating the autocrine and paracrine secretion of melatonin [4,7,23]. The synthesis and secretion of melatonin are closely interconnected with the growth patterns of cashmere in goats. Pinealectomy induced significant morphometric and biochemical alterations in the dorsal, abdominal, and thoracic skin, including reducing epidermal and dermal thickness, as well as decreasing the number of dermal papillae and hair follicles. The administration of melatonin to pinealectomized rats substantially ameliorated these abnormalities across all examined body regions [24]. Therefore, further research and comprehensive reviews on the effects of melatonin on skin hair follicles are warranted.

Recent research has uncovered that the endocrine hormone melatonin, along with various critical genes and signaling pathways, such as Wnt/β-catenin, BMP (bone morphogenetic protein), and FGF5 (fibroblast growth factor 5), plays a significant role in regulating the growth and development of hair follicles [25,26,27]. Numerous studies have concentrated on how non-coding RNAs regulate the cyclic growth of hair follicles, enabled by the extensive use of multi-omics data. In these studies, researchers also analyzed the differential expression patterns of target genes, microRNAs (miRNAs), and long non-coding RNAs (lncRNAs) across the anagen, catagen, and telogen phases of hair follicles [28,29]. Additionally, a growing body of research in recent years has highlighted that disturbances in the gut microbiome or gut health profoundly influence the skin barrier [30,31]. Studies have shown that Bifidobacterium longum increases levels of fecal and serum indole-3-carbaldehyde, modulates the composition of the gut microbiota, and alleviates atopic dermatitis through the gut–skin axis [31]. This review comprehensively addresses the mechanisms underlying the anabolic system of melatonin across various tissues (pineal gland, gut, and skin), how melatonin regulates hair follicle growth and development through various pathways (Wnt, BMP, miRNAs, lncRNAs, gut–skin axis), and the advancements in melatonin applications in cashmere goat production. This review elucidates the regulatory mechanisms of cyclic hair follicle growth, enhances strategies to improve fleece yield and quality in economically valuable animals, and advances therapeutic approaches for human hair follicle disorders.

## 2. Melatonin Secretion and Synthesis

### 2.1. Pineal Gland Interacts with SCN to Produce Melatonin

**Figure 1:**
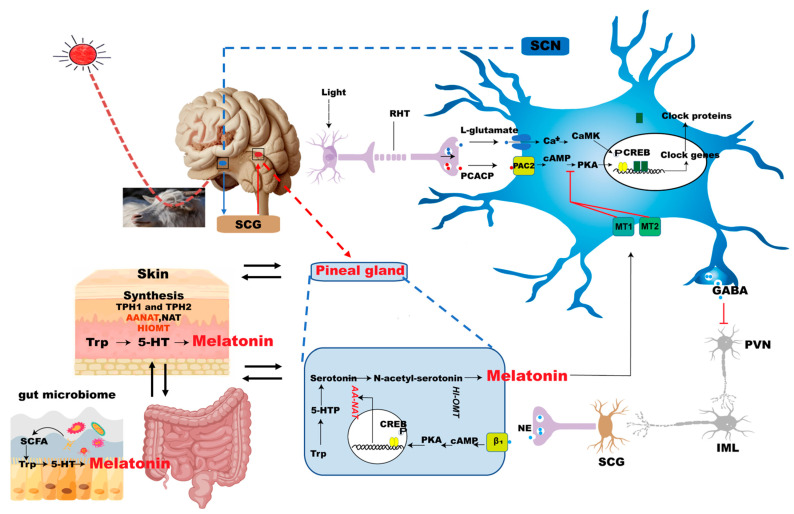
Interaction of the suprachiasmatic nuclei (SCN), pineal gland, skin, and gut microbiome with melatonin. Rhythmic pattern of clock gene expression reflected in the circadian activity of SCN cells. Light-activated melanopsin-expressing photosensitive retinal ganglion cells that project to SCN through the RHT. RHT axons release glutamate and PCACP, while PCACP mediates the expression of the clock gene in the SCN [40,51]. The SCN contains GABA neurons that send a direct inhibitory projection to neurons in the paraventricular nucleus of the hypothalamus (PVN) [41,52]. These PVN neurons activate preganglionic neurons in the intermediate lateral cell column (IML), and the IML activates melatonin secretion from the pineal gland via the superior cervical ganglion (SCG) [45,53]. Norepinephrine acts through beta-1 (β_1_) receptors to increase the activity of arylalkylamine *N*-acetyltransferase (AA-NAT), a key enzyme in the synthesis of melatonin [43,53,54]. Circulating melatonin inhibits the sleep-promoting activation of the SCN and resets the circadian pacemaker through melatonin MT_1_ and MT_2_ receptors [44,55,56]. Melatonin is synthesized in the skin through tryptophan (Trp), and melatonin has a complete anabolic system there. Short-chain fatty acids (SCFAs), metabolites of the gut microbiome, promote the synthesis of the melatonin precursors 5-HT and Trp. SCFA, short-chain fatty acid, Ca^2+^, calcium, CaMK, calmodulin kinase; CREB, cAMP responsive element binding protein; 5-HTP, 5-hydroxytryptophan; HI-OMT, hydroxyl indol-O-methyltransferase; NE, norepinephrine; NO, nitric oxide; PAC2, pituitary adenylate cyclase activating polypeptide receptor 2; PKA, protein kinase A.

In mammals, the suprachiasmatic nucleus (SCN) of the hypothalamus acts as the central circadian clock, regulating intrinsic circadian physiological rhythms to the surrounding 24 h light–dark cycle, allowing profound cyclical changes in physiological and behavioral states between high- and low-activity states [32]. The SCN synchronizes circadian physiological and behavioral rhythms, including sleep and wakefulness, temperature, feeding, and the neuroendocrine and autonomic effects, to the 24 h periodicity, thus coordinating the optimal internal temporal sequence [33]. The circadian rhythm regulating effect of the SCN on the organism is mainly influenced by the stimulation of light in domestic animals [34,35]. Light inhibits melatonin synthesis in addition to regulating SCN, the neural output signal generated by SCN that induces melatonin synthesis in the pineal gland at night [36] (Figure 1).

The pineal gland and the SCN of the hypothalamus, through the sympathetic neurons of the superior cervical ganglion (SCG), interact to form the body’s biological clock, with melatonin secreted by the pineal gland playing a key role [37,38]. The SCN acts as brain’s central pacemaker, synchronizing circadian physiological and behavioral rhythms, including full asleep, wake up, temperature, hunger, and neuroendocrine effects [39]. The retinohypothalamic tract (RHT) regulates SCN oscillations and melatonin secretion by transmitting light signals from retinal inputs; RHT comprises axons of light sensitive retinal ganglion cells that respond to short-wavelength light by expressing melanopsin [40]. RHT axons release L-glutamate and pituitary adenylyl cyclase-activating polypeptide (PCACP), which regulates the expression of SCN internal clock genes; this effect can reset the circadian rhythm and adjust the light synchronization of the SCN [41]. SCN utilizes γ-aminobutyric acid (GABA) as its primary neurotransmitter to directly inhibit a group of neurons in the paraventricular nucleus (PVN) that project to sympathetic neurons innervating SCG [42]. During the light phase of the photoperiod, enhanced SCN activity leads to a reduced sympathetic activation of PVN output to the pineal gland via the SCG, while a decreased release of norepinephrine leads to reduced pineal arylalkyl *N*-acetyltransferase (AANAT) activity and melatonin secretion [43]. During the dark phase of the photoperiod, the pineal gland secretes more melatonin; circulating melatonin acts through melatonin (MT1 and MT2) receptors to inhibit the activation of SCN (sleep promoting), and resets the circadian pacemaker [44] (Figure 1).

The main synthetic site of melatonin is the pineal gland, which can transmit the effect of SCN on the circadian rhythm of the body. Melatonin synthesis undergoes four enzymatic reactions starting from tryptophan [45]. First, tryptophan is hydroxylated by tryptophan hydroxylase (TPH) to form 5-hydroxytryptophan; then, 5-hydroxytryptophan is decarboxylated by aromatic amino acid decarboxylase (AADC) to form serotonin. Next, serotonin is converted to *N*-acetyl-serotonin by AA-NAT [46], and, finally, *N*-acetylserotonin is catalyzed to melatonin by acetylserotonin O-methyltransferase (ASMT) [47]. AANAT is a major rate-limiting enzyme in melatonin synthesis; cAMP-response element-binding (CREB) is a vital transcription factor of AANAT and its phosphorylation (p-CREB) regulates AANAT expression [48]. The SCG secretion of norepinephrine acts on adrenergic receptors to trigger cyclic adenosine monophosphate (cAMP) production, activating p-CREB and promoting AA-NAT activity [49,50]. Consequently, melatonin secretion is regulated by the photoperiod, and many sympathetic neurons participate in this regulation pathway, including SCN, SCG, and PVN [34] (Figure 1).

### 2.2. Melatonin Synthesis from the Skin

**Figure 2:**
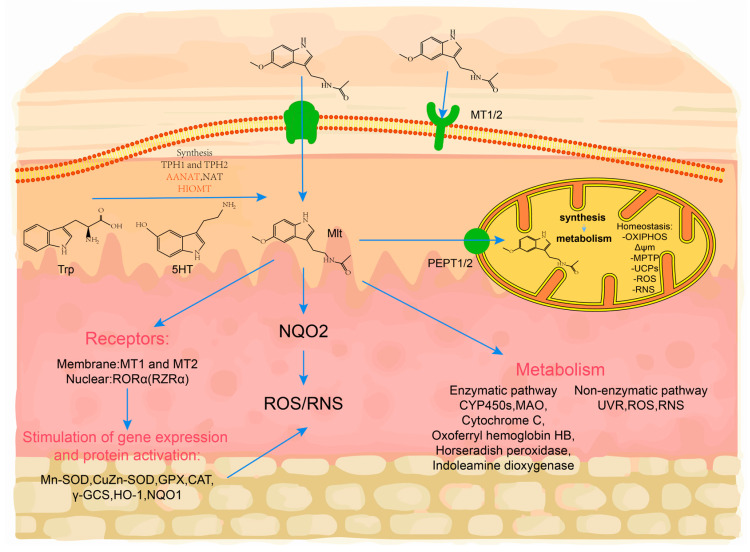
Melatonin production in the skin and the mechanism it protects the skin. Melatonin is generated from tryptophan (Trp) in multiple steps and can be metabolized via a range of enzymes and non-enzymatic mechanisms. Melatonin functions indirectly through membrane and nuclear receptors (MT1, MT2, and RORα) to stimulate gene expression and antioxidant enzyme activity, regulates quinone reductase type 2 (NQO2) activity, and directly and indirectly scavenges reactive oxygen and nitric species, resulting in reduced oxidative stress-induced cell damage. Melatonin transfer to mitochondria has been revealed to be mediated by the peptide transporter PEPT1/2 [65], which increases the capacity of the mitochondrial membrane potential by stimulating the uncoupling proteins (UCPs) and inhibiting the mitochondrial permeability transition pore (MPTP). As a consequence, oxidative phosphorylation (OXPHOS) produces more ATP. Its local metabolism and production of Trp, 5-TH, and melatonin will also affect these effects. NQO1, quinone reductase type 2, RNS, reactive nitrogen species, AFMK, *N*^1^-acetyl-*N*^2^-formyl-5-methoxykynuramine, AMK, *N*^1^-acetyl-5-methoxykynuramine.

Initial studies on Syrian golden hamsters found that the skin AANAT enzyme converts 5-hydroxytryptamine (5-HT) to melatonin. Subsequent investigations have revealed the presence of essential precursor molecules for melatonin biosynthesis within mammalian skin tissue, including the critical enzymes AANAT, ASMT, and TPH [57]. Notably, these findings demonstrate the existence of a self-sustaining melatonin synthesis pathway in cutaneous cell populations across mammalian species, including rodent models. The complete biosynthetic machinery encompasses three functional levels: (1) the transcriptional activation of related genes, (2) the translation of corresponding enzymatic proteins, and (3) the maintenance of catalytic activity for AANAT, ASMT, and TPH within cutaneous microenvironments [58,59]. As previously mentioned, tryptophan is an essential amino acid for melatonin synthesis, and the hydroxylation of tryptophan leads to 5-HT. The rate-limiting enzymes of melatonin are AANAT or ASMT, which produce parallel circadian rhythms to melatonin in the skin, and they are photoinhibited just like melatonin [60]. In addition, the skin synthesizes (6R)-L-erythro-5,6,7,8-tetrahydrobiopterin (6-BH4), a cofactor of TPH [61]. Melatonin concentrations in mouse skin, mouse hair follicles, and human scalp hair follicles, in terms of organ culture, far exceed their serum melatonin concentrations [62]. MT2 and retinoid orphan receptor alpha (RoRα) were detected in mouse skin, fluctuated in a hair cycle-dependent manner, and were maximal during apoptosis-driven follicular regression [62] (Figure 2). Thus, the skin hair follicle is both an important extrapineal melatonin synthesizing tissue and a prominent peripheral melatonin targeting tissue. Melatonin affects the hair growth cycle by regulating the cyclic activity of hair follicles.

Melatonin synthesis in the skin is not limited to keratinocytes but is also prominently localized in mitochondria. Mitochondria, as ancient endosymbionts, retain the evolutionary conserved ability to synthesize melatonin from serotonin via the enzymatic actions of AANAT and ASMT [63]. In skin cells, mitochondrial melatonin synthesis occurs independently of photoperiodic regulation and acts as a first-line defense against oxidative stress. This locally synthesized melatonin directly scavenges reactive oxygen species (ROS) within mitochondria, stabilizes the mitochondrial membrane potential, and enhances ATP production by stimulating uncoupling proteins (UCPs) and inhibiting the mitochondrial permeability transition pore (MPTP) (Figure 2). These mechanisms are critical for maintaining redox homeostasis in hair follicle stem cells and dermal papilla cells, thereby supporting their proliferative and anti-apoptotic functions [63,64].

### 2.3. Melatonin Synthesis from Gut Microbiome

Early studies found that melatonin synthesis was not limited to pineal cells but was also synthesized in the gastrointestinal tissues, and the content of melatonin in the gastrointestinal tract and serum increased significantly after feeding animals [66,67]. Recent studies have found that the gut microbiome promotes melatonin synthesis through multiple pathways [68,69,70]. Metabolites of the gut microbiome, SCFAs, especially butyric acid, and the tryptophan metabolite indole promote the production of 5-HT, a precursor of melatonin synthesis [70]. The enrichment of the probiotics *Akkermansia* and *Bifidobacterium* promotes higher levels of melatonin production by gut microbes [69]. Melatonin levels were reduced in the serum of antibiotic-treated and germ-free mice [68]. *Lactobacillus reuteri* (L. R) and *Escherichia coli* (*E. coli*) enhance AANAT expression while suppressing melatonin synthesis via the TLR2/4-MyD88-NF-κB pathway. MyD88 deletion in colonic epithelium abolishes the microbiota-mediated regulation of melatonin production [57]. Dietary fiber supplementation to backup sows increased the abundance of SCFA-producing microorganisms, promoted elevated serum 5-HT, and up-regulated TPH1 mRNA levels [71] (Figure 1). In addition, studies have found that gut microbiome-encoded sulfate esterase promotes serotonin and melatonin synthesis, and the probiotic supplementation of zebrafish increases the abundance of melatonin receptor transcripts and proteins (melatonin receptor 1) [72,73]. Therefore, melatonin anabolism is regulated by the gut microbiome and its metabolites via a variety of mechanisms.

## 3. Laws and Regulation Mechanisms of Cashmere Growth

**Figure 3:**
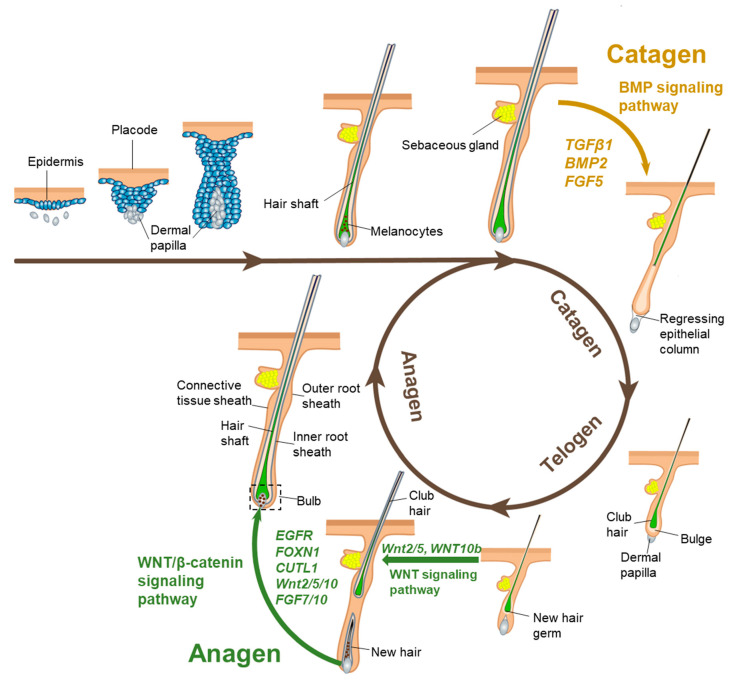
The hair cycle growth goes through anagen, catagen, and telogen. Each period is regulated by different signaling pathways, including WNT and BMP signaling pathways. Starting with the initial postnatal anagen, when the hair shaft begins to grow and poking through the skin’s surface, the stages of the hair cycle are shown. Apoptosis and regress occur in the lower two-thirds of follicles during the destructive (catagen) phase, which occurs simultaneously in follicle progression. When a critical threshold of activating factors is achieved, the dermal papilla is brought to rest below the bulge stem cell compartment, and the stem cells are triggered to regenerate hair after the resting (telogen) phase.

Cashmere grows seasonally and periodically. After the summer solstice, as daylight hours decrease, the growth rate of cashmere progressively accelerates; following the winter solstice, as daylight hours increase, the growth rate of cashmere gradually slows and eventually halts [74]. This growth phase corresponds to the anagen stage of the hair follicle cycle. As the photoperiod increases, SHFs transition into the catagen phase, which subsequently culminates in the telogen phase. During the telogen stage, the hair is easily shed, allowing for the harvesting of cashmere, after which the hair follicle enters a new cycle [74]. Hair follicle development is categorized into three main stages—anagen, catagen, and telogen—based on changes in follicle structure and hair shaft growth [75]. During the anagen phase, hair follicle cells exhibit heightened activity in proliferation and differentiation, marking the most critical period for hair shaft growth [76]; the activity of SHF is at its strongest, with the division activity of hair follicle (HF) cells being vigorous, the volume of hair balls being large, and the cashmere growth rate being fast. The catagen phase represents the primary period of atrophy in the circulating portion of the hair follicle; in this phase, the proliferative activity of the hair follicle declines, the matrix (Mx) cells undergo terminal differentiation, apoptosis occurs rapidly, and the hair shaft stops growing [76]. In the telogen phase, the circulatory structure of the hair follicle is fully absent; most hair follicle cells stop proliferating and differentiating, and the hair shaft gradually sheds as growth ceases [76] (Figure 3).

This cyclic mechanism, governed by the pineal and pituitary glands, is regulated by a photoperiod that consequently affects mammalian coat growth. Light suppresses pineal activity, whereas darkness enhances it [77]. Melatonin levels in the blood, secreted by the pineal gland, exhibit cyclic fluctuations in response to changes in day length [78]. Melatonin functions as a critical neuroendocrine regulator in goats, where coat phenotypes and reproductive changes are governed by photoperiodic effects on melatonin secretion. Extended periods of light inhibit melatonin synthesis, and this inhibition diminishes as daylight hours decrease. Rising melatonin levels and sharply falling prolactin levels trigger the onset of cashmere growth. Pinealectomy in voles abolishes the effects of short daylight on coat growth, indicating that the regulation of coat growth by light is mediated through melatonin secretion from the pineal gland [79]. Studies have shown that the center of secondary hair follicle growth initiation in velvet goats resides in hair papilla cells, and varying concentrations of melatonin promote hair papilla cell proliferation, with 300 pg/mL being the most effective, and mechanistically, melatonin up-regulates the expression of *Wnt10b*, a key gene in the Wnt pathway [80] (Figure 3). Furthermore, studies have demonstrated that exosomes from SHF regulate the growth and development of hair follicle stem cells (HFSCs), and that 500 pg/mL of melatonin significantly up-regulates the expression of marker proteins in these exosomes, which indirectly enhances the expression of key genes in hair follicle stem cells [81]. Previous studies have demonstrated that melatonin accelerates cashmere fiber germination, increases fiber length and density, enhances overall production, and reduces fiber fineness [82]. An experiment involving the administration of melatonin to velvet goats in Inner Mongolia in April and June effectively synchronized the melatonin-induced velvet growth phase with the natural growth cycle, thereby extending the cashmere growth period [83]. The underlying regulatory mechanism suggests that melatonin accelerates the activation of SHFs, prompting previously inactive follicles to re-enter the growth phase, thus promoting cashmere production [82]. Therefore, melatonin plays a crucial role in the proliferation and differentiation of secondary hair follicle stem cells in the skin.

Hair growth and cycling is also regulated by integrating systemic and locally produced hormones. Melatonin, prolactin, and pituitary–thyroid hormones each play distinct yet interconnected roles in this process. In the melatonin–thyroid axis, melatonin’s circadian influence may enhance thyroid hormone efficacy by aligning receptor expression with optimal growth phases. Conversely, thyroxine’s modulation of clock genes could amplify melatonin’s anti-oxidative and pigmentation effects. In prolactin–thyroid cross-regulation, TRH stimulates both thyroid hormones and prolactin, creating a dual role. While thyroid hormones promote anagen, prolactin counteracts this by inducing catagen, suggesting a feedback loop to prevent unchecked growth [84,85]. The dysregulation of these hormones manifests in hair disorders. For example, hypothyroidism leads to telogen effluvium, while hyperprolactinemia causes hair thinning [86]. Melatonin deficiency may accelerate graying and disrupt circadian growth cycles [87]. Therapeutic strategies targeting these pathways—such as TRH antagonists for hyperprolactinemia or topical thyromimetics for alopecia—could restore follicular homeostasis. Melatonin, prolactin, and pituitary–thyroid hormones collectively orchestrate hair growth through circadian regulation, mitochondrial activation, and catagen–anagen balance. Their interconnected signaling underscores the HF’s role as a neuroendocrine hub, offering novel targets for treating hair loss and hypertrichosis.

Sex-specific differences in melatonin synthesis are observed in cashmere goats. Fe-males exhibit higher melatonin levels during the breeding season. This difference is at-tributed to the influence of sex hormones, such as estrogen, which can enhance the activity of the pineal gland and increase melatonin production [83]. Additionally, the seasonal variation in melatonin levels is more pronounced in females, likely due to their reproductive cycles and the need for the precise timing of seasonal behaviors such as breeding and lactation [7]. These differences may influence seasonal cashmere growth patterns, with females showing more synchronized follicle activation.

## 4. Hair Follicles Growth and Development

### 4.1. Mechanics of Hair Follicle Formation and Periodic Growth

Numerous interconnected signaling pathways regulate the growth and development of hair follicles. During the fetal period of mice, the interaction between the epidermis and dermis results in the morphogenesis of hair follicles [88]. Signals from the dermal cells induce the epidermis to form hair buds; the hair buds release factors that induce the dermal fibroblasts to form the hair papillae and the hair papillae release a signal that encourages the proliferation and differentiation of the epithelial cells into a complete follicular structure [88,89]. From genesis to maturation, mammalian hair follicles are classified into three stages: induction, organ creation, and cell differentiation [90]. Hair follicle genesis and maturation are controlled by signaling factors in the WNT pathway, which is thought to be the master switch of hair follicle development [91]; the interaction of inducible factors *Wnt10b* and inhibitory *DKK4* in the WNT pathway directly affects the formation of hairy substrate [92] (Figure 3). The intermediate and late phases of hair substrate production are heavily regulated by the EDAR/NF-κB signaling pathway. In cashmere goats, the suppression of EDAR caused aberrant PHF growth, which resulted in hairlessness on the head [93]. Following the creation of the hair substrate, signaling molecules are produced to cause dermal fibroblasts to develop into hair papillae. These papillae then encourage the proliferation and differentiation of epithelial cells, resulting in the production of the inner root sheath and hair shaft, and ultimately the follicular organ. The inner root sheath and hair stem cell differentiation are the primary components of the hair follicle cells’ differentiation stage. Factors such as *Gata3*, *EGFR*, *FOXN1*, and transcription regulatory factor *CUTL1* govern the expression of keratinocytes in the inner root sheath. The expression of *LEF-1*, *HOXC13*, and *FOXN1* is required for hair stem precursor cell development in BMP signaling [94] (Figure 3). It has been demonstrated that Noggin-modified BMP4/BMPR-IA signaling promotes hair follicle-initiated growth, with follicular papillae entering the thriving phase and beginning normal proliferation and differentiation [95,96,97]. Once in the flourishing phase, hair follicle papillae start to divide and differentiate regularly. In order to stimulate the growth of hair follicles, hair papilla cells are able to create a lot of growth signals, including *Wnt2/5/10*, *FGF7/10*, and *KGF* [97] (Figure 3). The hair papilla ceases to divide during the regression phase; growth maintenance factors like *FGF* and *VEGF* are expressed less frequently, and inhibitory factors like *TGFβ1*, *BMP2*, and *FGF5* are gradually expressed more frequently [98] (Figure 3). The hair follicle enters the resting phase, the hair bulb continues to atrophy, and the high expression of *FGF18* and *BMP6* keeps the follicular stem cells perpetually in a resting state. In conclusion, the genesis, development, and cycle growth of hair follicles are governed by a series of signaling pathways and related genes.

### 4.2. Regularity of Hair Follicle Development in Young Cashmere Goats

**Figure 4:**
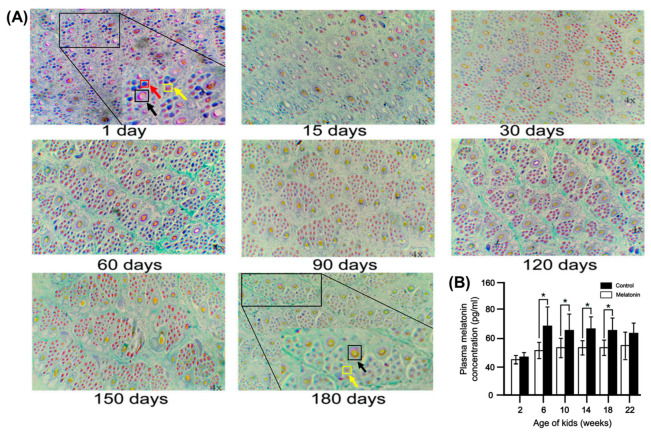
(**A**) Morphological changes in PHF and SHF in cashmere goat kids from 0 to 180 days. Skin samples were fixed in 4% paraformaldehyde, sectioned, and stained using the Sacpic method (Safranin O and Picric acid). Images were acquired under a light microscope (Nikon Eclipse E100, 40× magnification). PHF (black arrow), immature SHF (red arrow), and mature SHF (yellow arrow) are indicated [99]. (**B**) Serum melatonin levels (pg/mL) during postnatal development [7], * *p* < 0.05.

Hair follicles in cashmere goats are divided into two categories (PHF and SHF) based on their structural morphology. Our previous study indicated that the PHFs (black arrow) of Albas goat kids were fully developed within 1 month after birth, there were more underdeveloped SHFs (red arrow) within 4 months after birth, and the SHF were fully developed at 5 to 6 months of age (yellow arrow) [99] (Figure 4); this study also found that *FGF2*, *FGF21*, and *BMP7* were significantly higher at 3 months of age than at 1 and 6 months of age. The results also indicated that there is a window to regulate SHF traits and cashmere quality in lambs from birth to 6 months of age, and that the traits of the hair follicles at 6 months of age is an important predictor of cashmere quality in adult cashmere goats [99] (Figure 4A). Notably, plasma melatonin also peaked during this period (Figure 4B). A recent study analyzed cashmere goat skin transcriptome data from Inner Mongolia for 12 months using weighted gene co-expression network analysis (WGCNA); the results showed that the WNT signaling pathway plays an important role in the development of the hair follicle. Furthermore, 10 important genes associated with the hair follicle cycle, including *WIF1*, *WNT11*, *BAMBI*, *FZD10*, *NKD1*, *LEF1*, *CCND3*, *E2F3*, and *CDC6*, were identified and reached relative peak expression at the age of 3 and 4 months, suggesting that these genes play an important role in the regulation of follicular ontogenesis and development [100].

### 4.3. Law of Hair Follicle Development in Adult Cashmere Goats

Hair follicle cyclic changes are mainly formed by the interaction of epithelial and dermal cells; after the primary and SHFs are fully developed in postnatal lambs, they then begin to continuously undergo a cycle of anagen, anagen, and resting phases of growth, and the cyclic cycle of the hair follicle is closely related to light exposure [101]. The growth cycle of cashmere originates from the growth cycle of the secondary hair follicle. Taking the Inner Mongolian cashmere goat as an example, April is the shedding period of the cashmere, and, following this, the SHFs begin to be activated; after the summer solstice, there is a shift into the anagen period, and the secondary hair follicle outer root sheath cells begin downward extension, and the hair follicle activity rebuilding begins to gradually increase. From August to September, the hair follicle structure gradually completes, the number of follicles significantly increases, and then there is a shift into the thriving period. In October, the hair follicle cells stop dividing, the hair papillae gradually atrophy, and the roots of the hair begin to move upward; in December, hair follicle activity begins to gradually reduce, entering the regression period, and the hair root rises to the sebaceous glands and no longer changes until 1–3 months into the next year [83].

### 4.4. Advances in the Melatonin-Regulated Growth and Development of Hair Follicles

Melatonin’s significance in increasing the output of cashmere goat hair has been widely established for decades, and research on melatonin-mediated hair follicle growth has seen numerous new advances in recent years. In an in vitro study of an SHF culture system of cashmere goats, it was demonstrated that exposure to 500 ng/L melatonin promotes the multiplication of hair follicle stem cells, resulting in longer cashmere hair shafts [102]. Subsequent research discovered that 500 ng/L of melatonin generated a correspondingly strong Catenin beta-1 (CTNNB1) response, prolonging exposure to 72 h. More CTNNB1 entered the nucleus, activating transcription factor 4/lymphoenhancer binding factor 1. It also increased the expression of the proliferation-related genes *C-MYC*, *C-JUN*, and *CYCLIND1* [103]. A melatonin regulatory network of lncRNA (circRNA)-miRNA-mRNA was constructed by a study based on the whole transcriptome sequencing analysis of goat skin. It was discovered that miR-211, which is centered on miRNA-211, regulated the expression of several genes linked to hair follicle development, including *KRTAP3-1*, *KRT36*, *COL6A3*, *FN1*, and *FGF* [8]. Furthermore, it was discovered that circMPP5 could absorb miR-211 and subsequently regulate *MAPK3* expression, which in turn directly up-regulated key genes in the MAPK pathway (genes such as *FGF2*, *FGF21*, *FGFR3*, and *MAPK3*) to affect the development of SHF [8]. Melatonin inhibited NFκB, AP-1 proteins, and the expression of the secretory senescence-associated phenotype (SASP) cytokines genes (*IL-1β*, *IL-6*, *MMP-9*, *MMP-27*, *CCL-21*, *CXCL-12*, *CXCL-14*, and *TIMP-1,2,3*) in the SHF of older cashmere goats, resulting in delayed skin aging, improved follicle survival, and increased secondary hair follicle count [104]. In summary, the current study found that melatonin regulated hair follicle growth and development via the CTNNB1, lncRNA (circRNA), miRNA, and MAPK pathways.

### 4.5. Melatonin Induced the Combination of the Cashmere-Growth and Non-Growth-Cashmere Periods in Adult Goats

Significant advances have been made in the application of melatonin to enhance cashmere production performance in cashmere goats. During the cashmere growth stage, melatonin induces an earlier onset of cashmere growth and appears to accelerate shedding, accompanied by a secondary cashmere production phase and altered initiation timing of the growth cycle. In a study involving two-year-old New Zealand cashmere goat ewes, melatonin implants (125 mg every two months) were administered at one-month intervals from May to August [105]. The results indicated that melatonin treatment significantly accelerated cashmere growth; however, early shedding was observed (Table 1) [105]. A trial with continuous melatonin implantation (18 mg) on December 11, February 1, and April 1 involved ten young (8-month-old) and ten adult Angora goats [106]. Results indicated that melatonin treatments advanced spring shedding in adult Angora goats rather than delaying it, with no significant effect observed in young Angora goats (Table 1) [106]. In a trial conducted from December (winter solstice) to June involving 16 Liaoning cashmere goats (with an average body weight of 30 kg at one year of age), melatonin implantation administered at a dosage of 2 mg/kg body weight every two months successfully extended the cashmere growth period by three months [107]. Variations in these findings are likely attributable to differences in melatonin dosage and duration, yet melatonin implantation consistently extended the cashmere growth period.

During the non-growth stage, research demonstrated that administering a single 18 mg melatonin implant to New Zealand cashmere goats on 29 September and 10 November induced twice-seasonal cashmere production, compared to the natural cycle of January growth and August shedding [108]. In a study with ten feral goats implanted with melatonin (1.86 mg/kg BW) in October for two consecutive years, there was an indication that melatonin significantly lengthened cashmere, inducing early growth and extending the growth phase (Table 2) [109]. Melatonin implantation in spring substantially increased cashmere length in both lactating and non-lactating goats, with cashmere thinning observed twice yearly [110]. Research on Australian cashmere goats revealed that beginning in July, three 18 mg melatonin implants (one-month release) induced an additional three months of cashmere growth, followed by four months of short-term growth after shedding. In contrast, control goats exhibited no cashmere growth from July to November (Table 2) [111].

Earlier studies contain certain limitations and inconsistencies. Our research suggests that in seasonal cashmere goats, melatonin treatment during the non-growth period may prevent early shedding by synchronizing induced cashmere growth with natural growth phases. Most Liaoning cashmere goats display extended, year-round cashmere growth traits. Both phased and year-round melatonin implantation effectively promotes cashmere growth in Liaoning cashmere goats. Adjusting melatonin implantation timing can effectively prevent premature cashmere shedding [112]. In Inner Mongolian cashmere goats, melatonin implantation performed twice during the non-growth period (in April and June) increased cashmere production by 34.5% and 16.2% in half-sibling and non-half-sibling goats, respectively, and raised maximum cashmere length by 21.3% and 17.3% in seasonal cashmere-growth goats. This model effectively induces both cashmere growth and non-growth phases in goats, promoting cashmere production without affecting growth or reproductive performance [83]. In conclusion, the species and administration time of melatonin treatment are critical for improving cashmere production, and future studies should concentrate on determining the appropriate dose and timing of melatonin implantation for various breeds.

**Table 2:** Comparison of buried melatonin trials during the cashmere non-growth period.

Authors	Animals	Methods	Results
Betteridge [113]	Feral goats	Implantation of melatonin (18 mg) before and after the vernal equinox.	Cashmere grows early, falls off in autumn; second growth falls off in March.
Litherland [108]	Cashmere goats	Implantation of melatonin (18 mg) in the non-cashmere growth phase.	Promoted early growth of cashmere, but most melatonin-treated groups cashmere began to shed at week 19 of melatonin implantation.
Welch [109]	Feral goats	Continuous melatonin (1.86 mg/kg body weight) implantation or subcutaneous injection for two years.	In the melatonin treatment group, there were two instances of cashmere growth and the length of cashmere increased significantly, while in the control group, cashmere only grew in autumn and winter.
O‘Neill [110]	Lactating and non-lactating goats	Implantation of melatonin (18 mg) on 16 October, 30 October, and 13 November or 25 November	Cashmere goats grew wool twice a year; the length of the cashmere increased significantly.
Nixon [114]	New Zealand cashmere bearing goats	Implantation of melatonin (18 mg) in the spring (September in the southern hemisphere).	The PHFs began to grow wool after 14 days of implantation and the secondary follicles began to grow cashmere after 28 days.
Kloren [111]	Australian cashmere goats	Melatonin (26 mg) was implanted from July to October and from January to April.	In the melatonin-implantation group, cashmere began to grow at the end of July until it was shed on 1 December, and in January of the following year, cashmere began a new round of growth until it was shed in June of the same year.
Wuliji [115]	Spanish goats	During the non-cashmere growth period, 3 mg was taken orally daily for 5 weeks or 18 mg was implanted every 6 weeks.	Implanting melatonin induced the early growth of cashmere and increased cashmere yield.

## 5. Mechanism of Melatonin Promoting the Hair Follicle Growth and Development

### 5.1. Melatonin Enhances the Development of Hair Follicles in Cashmere Goats by Improving the Body’s Antioxidant Capacity

**Figure 5:**
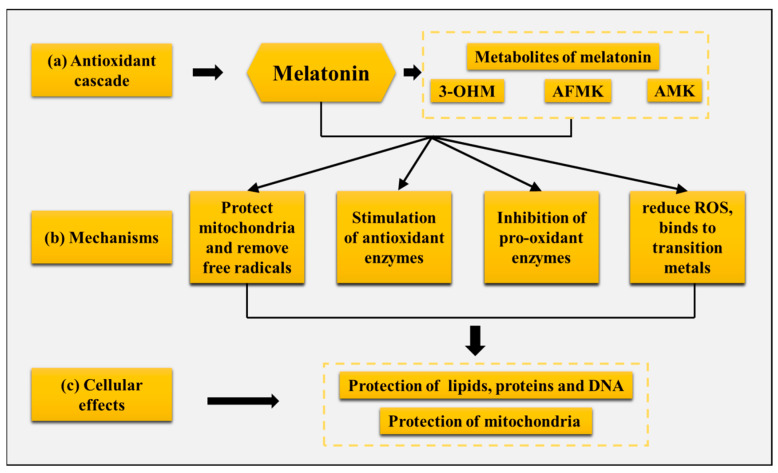
Melatonin’s antioxidant cascade and mechanisms of protection against oxidative damage. 3-OHM, 3-hydroxymelatonin, AFMK, *N*^1^-acetyl-*N*^2^-formyl-5-methoxykynuramine, AMK, *N*^1^-acetyl-5-methoxykynuramine.

Melatonin initially and primarily mainly served as a free radical scavenger and antioxidant in cyanobacteria roughly 3.5 to 3.2 billion years ago, neutralizing harmful oxygen compounds created during photosynthesis [116]. Over the next 3 billion years of melatonin evolution, its chemical structure has never changed, and the melatonin in cyanobacteria is structurally identical to that present in mammals today, with its original antioxidant function preserved [117,118]. Melatonin is very effective in reducing oxidative stress, it is highly soluble in lipids and partially soluble in water, and acts as an antioxidant in intracellular, body fluids, the aqueous environment of cell membranes and organelles [119,120]. Melatonin’s ability to cross physiological barriers and rapidly enter cells means that it is considered to be an ideal broad-spectrum antioxidant [121,122]: (a) it protects lipids, proteins, and DNA from oxidative damage [123,124,125], (b) it is highly concentrated in regions where free radicals are formed, such as in mitochondria, and melatonin synthesis is stimulated in the mitochondria [126,127], (c) both melatonin and its metabolites have a free radical scavenging function [128], (d) melatonin is also involved chelating cascade with metals, thereby reducing the formation of the aggressive reactive oxygen species (ROS) [129], and (e) melatonin induces the stimulation of the activity of some antioxidant enzymes [130] (Figure 5).

Melatonin’s antioxidant properties are deeply rooted in its mitochondrial actions. Beyond its systemic effects, melatonin synthesized in mitochondria directly neutralizes ROS and reactive nitrogen species (RNS), while its metabolites (e.g., AFMK and AMK) exhibit even greater radical-scavenging efficacy [131]. Furthermore, melatonin regulates mitochondrial quality control by promoting mitophagy, optimizing mitochondrial dynamics (fusion/fission) and stimulating biogenesis via the SIRT3-PGC1α pathway [132]. In cashmere goats, mitochondrial melatonin attenuates oxidative damage in secondary hair follicle (SHF) cells during the anagen-to-catagen transition, thereby preserving follicle integrity and extending the cashmere growth phase. This is evidenced by our findings of elevated SOD and GSH-Px activities in melatonin-treated goat skin, which correlate with reduced mitochondrial DNA damage and enhanced ATP synthesis [7].

Melatonin acts as a powerful antioxidant and free radical scavenger and is widely applied in livestock farming. In livestock production, ochratoxin A (OTA), a common fungal toxin produced by Aspergillus in feed, causes excessive intracellular oxidative stress, leading to organelle damage and even cell death, and melatonin administration was found to significantly ameliorate oxidative stress and apoptosis, which further protects the cell cycle and spindle formation in OTA-exposed oocytes [133]. Melatonin has been used as a vaccine adjuvant in pregnant sheep, and the injection of melatonin significantly improved the immune response to vaccination against *C. perfringens* [134]. In addition, ewes implanted with 18 or 36 mg melatonin 40 days before lambing had increased immunoglobulin concentrations in colostrum compared with the ewes not implanted [135]. Our previous study demonstrated that melatonin promotes morphogenesis and the maturation of SHFs in the skin by increasing the cashmere goat body’s antioxidant capacity, including significantly increasing superoxide dismutase (SOD) and glutathione peroxidase (GSH-Px) and the total antioxidant capacity (T-AOC) in skin and blood, reducing oxidative stress damage to the skin [82], and inhibiting apoptosis of hair follicle cells, including the down-regulation of the pro-apoptotic proteins Bax and caspase-3 and up-regulation of anti-apoptotic Bcl-2 expression in skin tissues [7]. The final result is to promote the morphogenesis and development of skin SHFs, increase the number of SHFs, reduce the fineness of cashmere, increase the cashmere yield, and improve the performance of cashmere goat production [7]. In addition, melatonin improves antioxidant activity and decreases ROS and RNS levels in SHFs in adult cashmere goats through the Keap1-Nrf2 pathway, including antioxidant genes (SOD-3, GPX-1, NFE2L2) and the nuclear factor protein (Nrf2), which were significantly up-regulated, while the Keap1 protein was significantly down-regulated [104]. Thus, melatonin improves the development of hair follicles in cashmere goats by increasing the body’s antioxidant capacity.

### 5.2. Melatonin Receptor Expression in the Hair Follicle

The periodic growth and development of skin hair is a complex process that involves the interaction of multiple cells and signaling pathways; skin is the main site for the physiological and biochemical action of melatonin, and melatonin receptors are present in epithelial cells, follicular stem cells, and papilla cells in human, mouse, and goat skin [136,137,138]. Among them, MT1 and MT2 receptors (formerly Mel1a and Mel1b) are membrane-bound G-protein-coupled receptors, originally identified as MT1 in retina and MT2 in the brains of chicken [139]; further studies identified the presence of melatonin binding sites MT1 and MT2 on peripheral tissues of the hamster, including the intestine, liver, kidney, lung, muscle, and heart [140]. Early studies have found that melatonin plays an important role in hair cycle control by down-regulating apoptosis and estrogen receptor-α expression and regulating MT2 and RORα expression [23]. In situ hybridization showed RORα expression in the SHFs of the hair shaft, inner root sheath, outer root sheath, medulla, and other target organs of the RORα receptor gene [141]. Subsequent studies in Inner Mongolian cashmere goats found that MT binds to the nuclear receptor RoRα on the dermal papillae to stimulate hair follicle development and promote cashmere growth, and the expression of RoRα in the skin tissue presents a periodic change, increasing from February, reaching a peak in April, and reaching a minimum in May; RoRα significantly affects the expression of β-catenin gene, a key gene for hair follicle development [142].

### 5.3. Melatonin–Gut–Skin Axis

The previous part of the article stated the existence of a complete synthetic and metabolic system of melatonin in both the gut and the skin, but little research has connected melatonin, the gut, and skin together. Gut microbiota were discovered to be the bridge between the immunological and neurological systems [143,144]. The gut microbiota directly or indirectly regulate the three major tryptophan (Trp) metabolic pathways that produce 5-HT, kynurenine (Kyn), and indole derivatives [145,146]. More than 90% of the body’s 5-HT is created in the gut, and as the main precursor substance for melatonin synthesis, 5-HT could be a crucial neurotransmitter connecting the melatonin–gut–skin axis [145]. In a recent sleep restriction (SR) trial, following melatonin administration, SR-induced skin damage improved, biological clock oscillations were strengthened, Bacteroides’ circadian rhythm was restored, and the rhythm of the propionic acid receptor GPR43 in skin was restored. According to this study, melatonin enhances the skin barrier by affecting the synthesis of SCFA, particularly propionic acid, and enhancing the circadian cycles of gut bacteria. SCFA are major metabolites of gut microbes that affect biological systems and their connections to gut–brain signaling pathways, including immune, endocrine, neural, and humoral channels [147]. Little research has been carried out regarding the melatonin–gut–skin axis; 5-HT and SCFA are the primary concerns for future studies on this topic.

## 6. Conclusions

This review synthesizes four decades of research, highlighting melatonin’s biosynthesis across tissues (pineal gland, skin, gut) and its regulatory roles in hair follicle dynamics. Key mechanisms include the following: (1) the activation of Wnt/β-catenin and BMP pathways to promote follicle proliferation; (2) antioxidant protection via Keap1-Nrf2 signaling; and (3) the modulation of non-coding RNAs (miR-211, circMPP5) to sustain hair cycle transitions. These insights advance strategies for improving cashmere production and inform therapeutic approaches for human alopecia.

## Figures and Tables

**Table 1:** Comparison of buried melatonin trials during the cashmere growth period.

Authors	Animals	Methods	Results
Mitchell [105]	New Zealand cashmere goat ewes	Implantations of melatonin in May, June, July and August, respectively, at the dose of 125 mg/ goat.	Cashmere grows early, growth rate significantly higher; second growth falls off in March, early shedding occurs.
Dicks [106]	Angora goats	Melatonin (18 mg) was implanted in cashmere goats in the late period of cashmere growth.	As a result, the cashmere fell off in advance and did not prolong the cashmere growth period.
Cong [107]	Liaoning cashmere goats	Three consecutive implantations of melatonin (2 mg/kg BW) at the end of December, February, and April.	The control group stopped growing in March, while the implant group continued to grow until June.
